# Impact of Virtual Imaging Technology on Film and Television Production Education of College Students Based on Deep Learning and Internet of Things

**DOI:** 10.3389/fpsyg.2021.766634

**Published:** 2022-03-30

**Authors:** Chengye Du, Chijiang Yu, Tingting Wang, Fengrui Zhang

**Affiliations:** ^1^School of Art of Articulate Speech, Sichuan University of Media and Communications, Chengdu, China; ^2^People’s Daily, Beijing, China; ^3^Department of Arts, Hoseo University, Asan, South Korea; ^4^Network Audio-Visual Culture Innovation Team, Hebei University, Baoding, China; ^5^Trinity College, University of Cambridge, Cambridge, United Kingdom

**Keywords:** deep learning, virtual reality technology, TAS scale, post-production, mental health of patients

## Abstract

More and more schools begin to design simulation technology based on virtual imaging technology (VIT) and virtual reality (VR) in their course contents. In particular, among these technical courses, there is a need to first strengthen the Film and Television Production (FTP) education in higher institutions. This article aims to study the impact of VRT, VR, and Internet of things (IoT) technology on FTP courses and audience psychology in higher institutions under the era of intelligent multimedia. How to use emerging VR technology to promote the psychological wellbeing of students or patients has become a new research direction, the exploration of which has a far-reaching significance for the applications of the related technologies. First, the principle and applications of VR and IoT technology are described. Thereon, the deep learning (DL)-based training model is used to analyze the postproduction (PP) of VR-based Sand Table game, and the function and effect of the designed game model are discussed. Subsequently, VR-based Sand Play Therapy (SPT) is applied to mentally ill patients to obtain its therapeutic effect. The results show that the designed VR-based Sand Table game model can be used to treat mentally ill patients and alleviate their negative psychological states. Meanwhile, the Test Anxiety Scale (TAS) scores prove the significant therapeutic effect of the designed game model on the mental problems of patients. Therefore, VR-based psychological SPT can be applied in the stress relief of students and the treatment of mentally ill patients, as well as alleviate their mental health problems. This research provides a new direction and some theoretical support for the application field of VR technology.

## Introduction

Under the modern socio-economic environment, even if material abundance has reached an unprecedented high level, living pressure of people, especially psychological pressure, has seen a record high level, causing all sorts of mental diseases, which is believed to be caused by the acceleration of the pace of work and life. For example, incomplete statistics show that the advent of the twenty-first century is accompanied by more than 800,000 suicide cases worldwide every year (Aline et al., 2018). In 2010 alone, approximately 222,300 Chinese have committed suicide. In 2016, the average suicide rate per 100,000 Chinese is estimated to be 7.8 males and 8.1 females, respectively. Globally, suicide has become the second leading cause of death for young people or the 15th leading lethal factor for people of all ages (Lew et al., 2021). In particular, test anxiety (TA) is a typical psychological reaction in the test process. It is inspired by the test situation, affected by personal experience, evaluation, personality, characteristics, and anxiety, as well as emotional pressure of subjects of passing the test. At the same time, TA shows some adverse psychological reactions. Therefore, this study focuses on how to utilize new virtual imaging technology (VIT) or virtual reality (VR) technology to create a digital or virtual environment for patients without direct physical contact, so that patients can express their hearts, emotions, and intrinsic feelings more freely to help formulate more targeted treatment strategies for mental diseases.

In recent decades, with the progress of science and technology, VR technology is seen broad application in traditional artificial methods to assist psychotherapy, thereby treating patients online without a prerequisite for a hospital visit (Rogoza et al., 2018; Wu et al., 2019; Wu Y. J. et al., 2020). For example, Liu et al. (2019) discussed the efficacy of progressive Sand Table Therapy (SPT) on the core symptoms and sleep management of preschoolers with mild to moderate autism spectrum disorder (ASD) and proved the effectiveness of SPT in autism treatment. Roesler (2019) studied the effect of SPT on intractable mental diseases under traditional treatment and found the potential of SPT in traumatic stress, disability, or language disorder treatment. SPT is a non-verbal method, especially suitable for children and adults with trauma, pain, disability, and transference problems (Roesler, 2019). Zhou et al. (2018) proposed to localize the SPT model by combining the symbolic meaning of Chinese classical cultural images. Veronica et al. (2018) provided standard treatment for post-traumatic stress disorder (PTSD), complex sadness, or adaptation according to the diagnosis of patients and the psychotherapy recommendations under traditional methods and VR technology. The above literature research proves that the Sand Table game can be used to treat mental diseases, which is a special pattern and word-based performance tool and is a relatively novel approach. Zhang et al. (2018) compared the effects of VR SPT and real SPT in psychological evaluation and treatment and proved their consistency. Nowadays, how to use VIT or VR technology to treat mental diseases and alleviate psychological stress has become a new medical research field. Specifically, many researchers have put forward effective treatment methods and VR technology for psychological stress. Artificial intelligence (AI) media and Internet of things (IoT) technology are seeing extensive applications, and VIT is penetrating all walks of life (Wu and Song, 2019; Yu, 2020). Therefore, the literature review indicates that VR technology, as a real-world simulation tool, can fabricate patients with a spiritual world customized to their needs. AI media is equal to media intelligence, that is, the media combines human intelligence with machines (computers) and reconstructs the whole process of news information production and dissemination based on AI technology. For example, combined with AI, mobile Internet (MI), big data, VR, and other new technologies, AI media includes intelligent media and think tank media. The integration of AI media and deep media technology will shift the crux of the existing media ecology from mass media to digital media in communication and life of people. Technologies, such as big data and algorithmic data processing, are hidden behind media communication and media education. In other words, digital data and hiding algorithms are intelligent mechanisms to manage user activities, behaviors, attention, content, information, and knowledge under new media and digital technology (Ptaszek, 2020). Therefore, related studies suggest that VR can also be combined with the IoT to provide personalized treatment services for patients by searching patient-related data over the network. AI media technology can aggregate and disseminate information, and then provide it to users indiscriminately according to the needs of users. In particular, “AI media” can filter and expand information, process it according to the needs of users, and then provide users with targeted information. Some experts believe that multimodal medical image fusion can obtain more comprehensive and better-quality images by integrating the complementary information of medical images, thereby providing more accurate data for clinical diagnosis and treatment. The new medical image fusion method using multi-scale edge-preserving decomposition and sparse representation can better preserve the details and structure information of the source image (Jiang et al., 2018). VIT refers to the construction of stereoscopic images in three-dimensional (3D) space through various technologies, such as augmented reality (AR) (Krichenbauer et al., 2018), VR (Parong and Mayer, 2018), and holographic display (Elbamby et al., 2018). The IoT (Soner et al., 2020) can collect real-time information through various devices and technologies, such as information sensors, radio frequency identification (RFID) (Seco and Jimenez, 2018), global positioning systems (GPS), infrared sensors, and laser scanners, by which things and people are connected through network access, and intelligent perception, identification, and management of things are realized. IoT technology is an intelligent management technology of physical goods and an extension of Internet technology (Li et al., 2018; Yuan and Wu, 2020). In different scientific fields, the abbreviation 3D is used flexibly and has multiple connotations, so it is easy to forget what it stands for in a specific context. The technical status of the generic term 3D is outlined in this study to help explain the importance of 3D in cartography (Claudia and Manfred, 2014). The cognitive representation of the learned map information is affected by the system distortion error (Doyle and Magor, 2018). Dividing the map surface into map elements of multiple regions, such as content-related linear symbols (street, river, and railway system) or additional artificial layers (coordinate grid), provides a novel pattern to help users reduce the distortion of their psychological representation. Thus, the relevant research helps to inspire a novel way to combine the Sand Table game with VR to provide a novel treatment for mentally ill patients. In recent years, the television industry has begun to use true-3D (automatic stereoscopic) displays as mass media. These modern displays can view dynamic and still images and depth illusions without additional equipment, such as 3D glasses. In these images, visual details can be distributed in different positions along the depth axis (Edler et al., 2015). In conclusion, SPT can well alleviate the mental diseases of patients of all ages, especially children and adults with trauma, pain, disability, and transference problems. VR SPT has shown the same good effect as real SPT. Therefore, the relevant research hereon further proves that the Sand Table game can be combined with VR to create a VR SPT model to break through the space limitation. Therefore, this article specifically studies the postproduction (PP) of the Sand Table game, improves on it, and tests its effect.

More concretely, the psychotherapeutic Sand Table game is taught to specific students through multimedia to alleviate their mental health problems. Psychological game films amplify the instability of ontology, remove the sovereign subject and its antidote, namely, the division between self and modern subjectivity, and encourage the client (students) to accept more complex, contradictory, and limited but broader forms of agency. Today’s era is witnessing the emergence of various psychotherapy methods and psychological hypotheses. In this study, the principle and processes of SPT are studied in detail, and VR technology is used to virtualize SPT, namely, VR-based SPT. Then, the Sarason Test Anxiety Scale (TAS) scores before and after treatment of VR-based SPT are comparatively analyzed to evaluate its therapeutic effect on mental problems. First, the “Introduction” section explains the research background and motivation, then expounds that a Sand Table game will be established through VR and VIT. The “PP of the Sand Table game and its therapeutic effect” section describes the relevant theoretical knowledge of Sand Table game, IoT technology, and VR technology VR and establishes a VR-based Sand Table game. Later, based on the designed Sand Table game under the IoT environment, the model usability is evaluated, along with its therapeutic effect on mentally ill patients. Thereon, the applicability and reliability of the designed Sand Table game are verified. It is hoped to provide some references and basis for psychotherapy and virtual educational courses. The content structure of this article is as follows: the “Introduction” section describes the concept and purpose of the Sand Table game, summarizes some functions of Sand Table game in relevant fields, then introduces the IoT technology and VR technology, as well as the mechanism to combine them, and further discusses the design process of virtual Sand Table game based on VR technology. This article briefly predicts the therapeutic effect of the proposed virtual Sand Table game on mentally ill patients and then establishes an agent action generation model for patients with the technical support of Phase Functioned Neural Network (PFNN). In particular, the proposed model is applied to mentally ill patients to explore the psychotherapy effect of the proposed virtual Sand Table game and corroborate its feasibility.

## Postproduction of the Sand Table Game and Its Therapeutic Effect

### Real Sand Table Game

Sand Table game is a psychotherapeutic method. The operation process is as follows: in a free and safe place, participants are allowed to build a scene out of their thoughts using the Sand Table game mold. This scene is believed to be an internal environment created based on their inner state. While immersed in the Sand Table game, participants shape the Sand Table through their initial ideas. Sand Table game involves active imagination technology, which can express human subconsciousness. It has been argued that the scenes created by individuals in Sand Table games help improve their personalization and complete personality. Sand Table game is the link between patients and therapists. Therapists can judge the symbolic meaning of the Sand Table image with concrete characteristics to try to understand the deepest subconscious thoughts of patients (Rajeswari et al., 2019).

**FIGURE 1:**
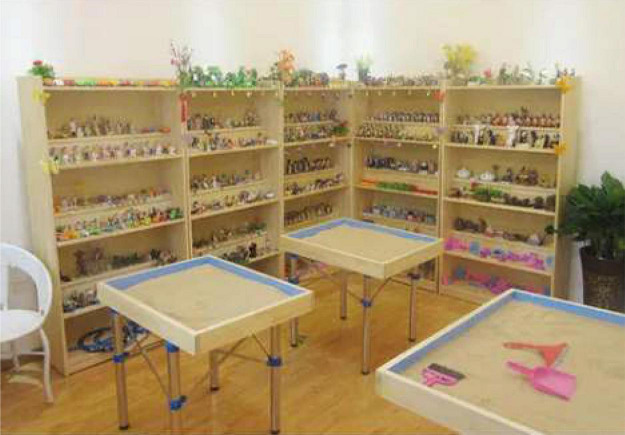
Sand Table game and treatment scene.

**FIGURE 2:**
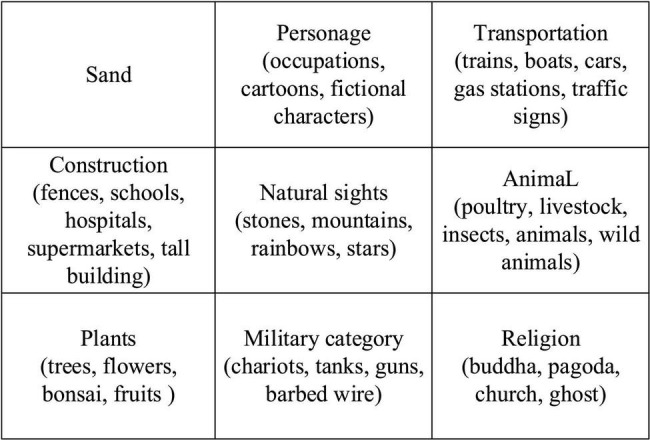
Sand Table game mold and essential materials.

Figure 1 shows the psychological Sand Table and treatment scene. Figure 2 displays the psychological Sand Table mold and its essential materials.

**FIGURE 3:**
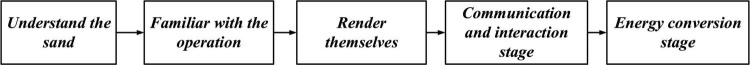
Psychological Sand Play Therapy (SPT) process.

As its name suggests, sand is the most basic material used as the placement foundation and background of other molds in SPT. Molds include 9 categories and 57 subcategories, which can be used to express various scenes and feelings at the life and psychological levels. Figure 3 illustrates that the process of psychological SPT (Punnett and Canfield, 2020).

a. Getting to know the Sand Table: the therapist establishes mutual trust with the patient, understands the situation of the patient, and induces the patient to pay attention to the Sand Table.

b. Familiarization of operations: the therapist leads the patient to get familiar with the Sand Table, guides the patient to experience the inner induction of the Sand Table, and then lets the patient get familiar with the mold.

c. Self-presentation: the patients are asked to use the water and sand in the Sand Table to shape their psychological pictures, during which the therapist is not allowed to interfere with. After the patient completes different shapes, the therapist needs to interpret the scene in the Sand Table to find the key to the mental problems of patients.

d. Communication and interaction: no verbal language is used for communication, and only the Sand Table and molds are used to express their emotions. During this period, there is no need for too much verbal communication, so that the emotions of patients can be unwittingly reflected.

e. Energy transformation: after the first four processes, the therapist and the patient have gained enough friendship and can get along harmoniously. At this time, the patients can shape the Sand Table through the cognition of their inner conflict and negative influence through the mold placement and the expression intention in the Sand Table scene, which transforms the subconscious of patients into consciousness. Thus, the therapist can interpret the inner feelings and emotions of patients through the Sand Table structure and expression of image representation characteristics (Rajeswari et al., 2019).

The principles of the Sand Table game are shown in Table 1.

**TABLE 1:** Principles for Sand Table game.

Serial number	Principle name	Specific precautions	Principle
1	Unconscious principle	Throughout the treatment process, patients are undisturbed and are supposed to be absorbed in their game unwittingly, without being informed of any specific purpose.	In the unconscious state, the feelings are fully exposed.
2	Symbolic principle	The therapist is supposed to have the whole process under perfect control.	Each time a different mold and Sand Table are chosen, their conversation will show the fluctuation of the patient’s inner situation.
3	Playfulness principle	It is particularly used to reveal the participants’ inner character, so it needs to be playful.	During the game, one’s own emotions will be amplified, external interference will be reduced, and true feelings will be revealed.
4	Common situation principle	The therapist is supposed to accompany the patients to empathize with them, let the patients share their feelings, act as the first listener, and finally find a cure.	Emotional resonance

Each Sand Table game can try to have multiple emotional themes, such as trauma theme and healing theme. Interpretation and analysis from multiple themes can change the Sand Table game constantly in the process, during which there is a need for the therapist to accurately grasp the psychological situation of the patients for timely communication and emotional outlet.

### Internet of Things Technology

**FIGURE 4:**
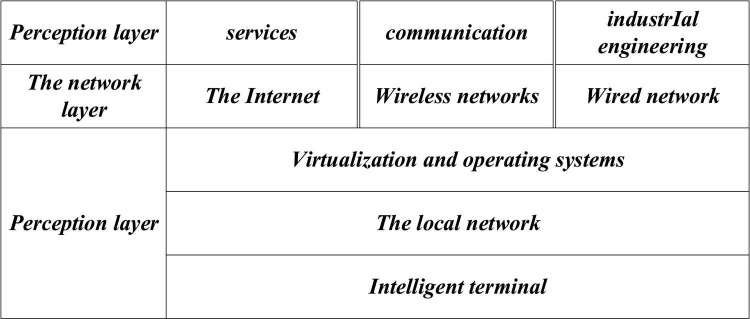
Basic architecture of Internet of things (IoT).

Internet of things technology has seen its birth in the Massachusetts Institute of Technology (MIT) in 1999. It is a new information service architecture based on Internet and RFID communication technology. The IoT can provide safe and reliable “goods” information through the Internet using the information technology infrastructure, thus creating an intelligent environment to identify and determine “goods” and promoting the information exchange within the supply chain (Wu and Wu, 2017; Zheng et al., 2018; Wu Y. J. et al., 2020). Figure 4 demonstrates the basic architecture of the IoT. The perception layer of the first layer is responsible for user service functions, such as communication or planning of industrial projects. The network layer is compatible with a diverse network structure, such as the Internet, wired network, and wireless network, while the perception layer of the bottom layer provides various service devices, such as virtualized operating systems (OS) and intelligent networking terminals, in the local network.

### Virtual Reality-Based Sand Table Game Design Process

The earliest concept of the virtual image in history dates back to *Sword of Damocles* by Ivan Sutherland in 1969. Since the *Sword of Damocles*, researchers have been trying to realize AR, VR, and mixed reality (MR). Realizing real-time 3D capture, reconstruction, and understanding of human beings and their world is the key technical engine to implement ubiquitous MR (Gilla et al., 2019; Shohei et al., 2019). Moreover, studies have shown that the Arcade machine “Sensorama” created by Morton Heilig in 1962 is the earliest research example of an Immersive (multisensory) VR (IVR) system (Laura et al., 2018; Sünger and Çankaya, 2019). VR is a comprehensive technology integrating computer technology, sensor technology, human psychology, and physiology. VR simulates the external environment through computer simulation systems. The main simulation objects include environment, skills, sensing equipment, and perception, thereby providing users with multi-information, 3D dynamic, and interactive simulation experience. So far, VR has been combined with IoT technology (Elbamby et al., 2018; Thies et al., 2018; Kilberg et al., 2020).

**FIGURE 5:**
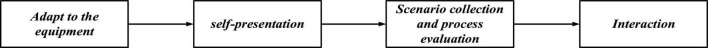
Virtual reality (VR)-based Sand Table game treatment process.

Virtual reality has three main characteristics, namely, immersion, interactivity, and imagination. Immersion refers to that the external environment simulated by the computer simulation system is very realistic. Users are completely immersed in the 3D VR environment, and it is difficult to distinguish the truth from the fake of the simulated environment. Everything in the virtual environment looks real, sounds real, and even smells real, which is exactly as immersive as the real world. Interactivity means that users can operate objects in the virtual world and get feedback. For example, users can grasp an object with their hands in the virtual world, recognize the shape of the object with their eyes, weigh an object with their hands, and the object can be easily manipulated and moved. Imagination means that the virtual world has greatly broadened the imagination of people in the real world. Under a VR environment, people can imagine a world or situations beyond the objective world. VR can be divided into four categories, namely, non-immersive VR, immersive VR, distributed VR system, and AR system. Figure 5 reveals the VR-based Sand Table game treatment process, and Table 2 indicates the specific explanation of the treatment process. VR technology is divided into the desktop VR (DVR) system, IVR system, AR system, and distributed VR system, of which the Sand Table game can lend itself best in the DVR system. DVR system is based on the ordinary computer platform. Usually, a personal computer (PC) or primary graphics computer workstation is used to construct the simulation environment, and the monitor visually displays the virtual environment for user observation. Users sit in front of the computer monitor, wear 3D glasses, and use devices, such as position tracker, data gloves, or 6-degree of freedom (DOF) 3D space mouse to manipulate the virtual scene and objects, browse the virtual world within 360 degrees, and play the Sand Table game on a virtual desktop.

**TABLE 2:** Specific steps of the treatment process.

Serial number	Name	Specific steps
1	Adaptive equipment	The therapist guides the patients to learn to operate VR equipment, get familiar with the operation, adapt to VR scenes and their impact on the senses, and start to build the simulation of the inner world on the Sand Table.
2	Self-realization	An absolutely private environment should be provided, which will not be disturbed by the outside world, where patients can reveal their feelings and build a Sand Table.
3	Process analysis and evaluation	The therapist will perform corresponding operations at the other end of the VR system. The therapist’s existence will be hidden from the patients, but the therapist can monitor the whole process and results in real time, and then analyze and improve them.
4	Communication and interaction	The therapist has a dialogue with the client. After analyzing the process and results, the consultant will communicate with the patient.

Virtual reality-based Sand Table game contains the characteristics and some rules of the real Sand Table game. However, thanks to VR technology, patients can be given more private space to reveal their intrinsic feelings, so that therapists can better analyze the real-time states of patients. The construction of the VR-based Sand Table scene is divided into the following parts: system interface, game interface, surface game layer, virtual model, and image of Sand Table, and optional scenes and settings.

**FIGURE 6:**
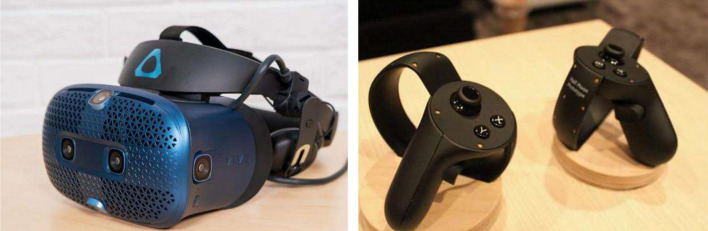
HTC VIVE wearable device.

HTC VIVE wearable device with two handles and the VR helmet is used in this study, as shown in Figure 6. HTC VIVE is developed using Unity development engine and network communication technology based on Network View.

The built-in system of HTC VIVE and Unity engine are used as the development tools. Hardware and software configurations are as follows: GTX2080 Graphics Card, Intel i7-7300 CPU, HDMI 1.4 video output, Windows 10 operating system, and C#.

**FIGURE 7:**
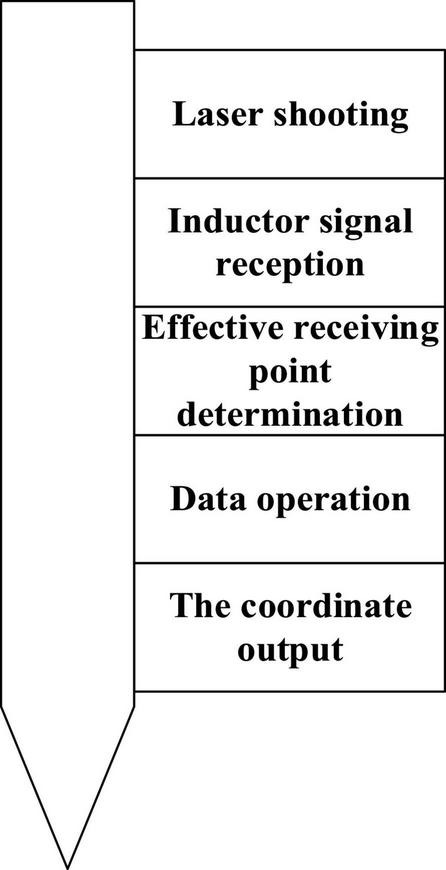
Positioning flowchart.

HTC VIVE system uses Lighthouse optical tracking technology. Lighthouse is composed of two base stations, and each base station has an infrared LED array and two rotating infrared laser transmitters whose rotating axes are perpendicular to each other, with a revolution of 10 ms/round. The working state of the base station is as follows: at the beginning of the cycle (a cycle of 20 ms), the infrared LED flashes; within 10 ms, the rotating laser of the *X*-axis sweeps the whole space, and the *Y*-axis does not emit light; within 10 ms, the rotating laser of the *Y*-axis sweeps the whole space, and the *X*-axis does not emit light. The Lighthouse base station valve under the high-speed camera has many photosensitive sensors installed on the head display and controller. After the LED of the base station flashes, the signal will be synchronized, and then, the photosensitive sensor can measure the time when the *X*-axis laser and *Y*-axis laser reach the sensor, respectively. This time is exactly the time when the *X*-axis and *Y*-axis laser turns to this specific angle to illuminate the sensor, so the *X*-axis and *Y*-axis angles of the sensor relative to the base station are known; the position of the photosensitive sensors distributed on the head display and controller is also known, so that the position and motion trajectory of the head display can be calculated through the position difference of each sensor. Lighthouse is a laser-based inside-out open-source positioning system. The open-source content includes the following three parts: (1) hardware: to be specific, the hardware design and implementation details of the hyper-real laser positioning system are introduced, involving circuits and embedded software. The components involved in the open-source are summarized and sorted out from the version of the principle verification machine of the hyper-real laser positioning system, and other contents irrelevant to the positioning characteristics are eliminated in the verification system design. The overall structure of the system is divided into two relatively independent parts, where the code chip is inserted, namely, the laser harness scanning lighthouse and the located equipment as the positioning beacon; (2) software: it is the hyper-real positioning equipment, including drivers and example programs of inertial measurement unit (IMU) and the light sensor. With these drivers, VR head-mounted display and solution providers can easily obtain the attitude and position information of the located equipment and the position and status of the lighthouse and then integrate them with the hyper-real positioning algorithm library. Third parties can integrate the hyper-real lighthouse positioning scheme into their own software development kit or application; (3) algorithms: hyper-reality (HR) directly provides a complete lighthouse positioning algorithm library, including all core algorithm modules: lighthouse scanning and IMU data analysis and processing, attitude solving algorithm, data fusion algorithm, double lighthouse fusion algorithm, and motion prediction algorithm. The algorithm library framework is completely based on a modular design, based on which developers can carry out algorithm research and customized development. This process can be divided into several parts as shown in Figure 7 (Qian et al., 2018).

**FIGURE 8:**
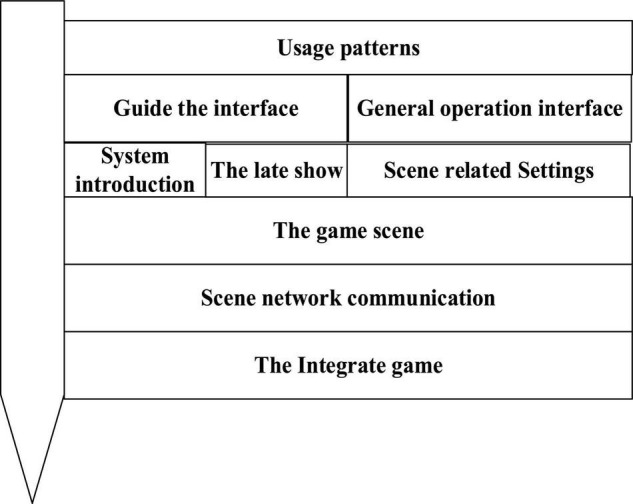
Sand Table game system design process.

Figure 8 shows the design flow of the Sand Table game system.

Sand Table game system design process is as follows: first, the game interface is designed, including the user guidance interface (guidance tutorial), general operation interface, and system introduction. Then, the game scene and related parameters are designed, including environment and props, as well as the acquisition mode, usage mode, and tutorial of these props; second, the network communication mode of the scene is designed; finally, all factors are integrated to form a complete game process.

The 3D reconstruction of the Sand Table game model is shown in Table 3.

**TABLE 3:** Three-dimensional (3D) reconstruction of Sand Table game model.

Serial number	Steps	Detailed steps
1	Data collection preprocessing	Real Sand Table and relevant materials are taken pictures, based upon which the size of Sand Table game model parts is measured.
2	Monomer modeling	The modeling proceeds from low to high accuracy components and the final Sand Table game model is created according to the calculated parameters and proportions.
3	Selection of materials and texture	Images are further processed, materials are arranged, and textures are pasted.
4	Model optimization	The model is simplified
5	Animation recording	Required test actions are performed

Ten professional psychological researchers are selected to conduct a Questionnaire Survey (QS) on the usability of designed Sand Table games, and the QS is scored from the following five aspects: (a) the beauty of the system interface: 1, beautiful; 2, generally beautiful; 3, not beautiful; (b) Is the guiding significance of the system interface is clear? 1, clear; 2, generally clear; 3, unclear; (c) the importance of system interface: 1, very important; 2, important; 3, unimportant; (d) Is Sand Table mold conceivable? 1, inconceivable; 2, appropriately conceivable; 3, conceivable; (e) Will the virtual scene affect the audience? 1, yes; 2, possible; 3, definitely not. The detailed questionnaire is presented in Table A1 in the appendix.

### The Influence of Virtual Reality-Based Sand Table Game on Audience Psychology

The state of anxiety is a psychological state between emotional anxiety and anxiety disorder. On the one hand, compared with anxiety disorder, its symptoms are lighter. On the other hand, the state of anxiety has obvious symptoms of emotional anxiety such as, easy irritability, annoyance, nervousness, and difficulty in sleeping or eating, which might also be accompanied by sleeping disorder and autonomic nerve disorder. Generally, the duration is short and can be recovered through self-regulation and self-suggestion.

Anxiety disorder, also known as anxiety neurosis, is a common neurosis in modern society. It can be divided into two forms: chronic (extensive) and acute anxiety (panic attack). The clinical manifestations are nervous and worry without clear objects, or restless sitting and standing, accompanied by autonomic nerve dysfunction symptoms. Some patients have anxiety disorder due to organic disease and brain injury. The incidence rate of anxiety disorders in China is also increasing yearly, and the incidence is becoming younger (such as college students, middle school students, and even some primary school students). Most commonly, a state of anxiety is seen in students due to TA that can lead to a series of problems, such as inattention, memory decline, affecting academic performance, and immunity decline. According to incomplete statistics, in China, college students with TA account for approximately 25% of the total college population; in other words, Chinese students are prone to mental problems due to tests (Chen, 2019).

Sarason TAS is compiled by professors of the *Psychology Department of the University of Washington* in 1987, including 37 items, and each item has two options of “yes” and “no.” Items 3, 15, 26, 27, 29, and 33, as well as answer “no” score 0 points, while other options and answer “yes” score 1. TAS itself has high reliability and validity (Snaith et al., 2018).

Then, 600 freshmen to junior students from Film and Television Production (FTP) and Multimedia majors in one university are selected to introduce the VR-based Sand Table game. A total of 124 participants are divided into experimental group and control group, and each group includes 30 men and 32 omen, averaging 21.14 years old. That is, there are 62 subjects in the experimental group which are randomly divided into 8 groups, with 6–9 people in one group; the control group also has 62 people, with 30 men and 32 women, with an average age of 21.52 years. Sand Table game experiment is conducted on the experimental group.

Furthermore, SPSS 22.0 (SPSS Inc., Chicago, IL, United States) is used for data processing and analysis, and the *t*-test is used for comparative analysis between the two groups, with an inspection level α = 0.05.

### Video Action Generation Model Based on Phase Functioned Neural Network

**FIGURE 9:**
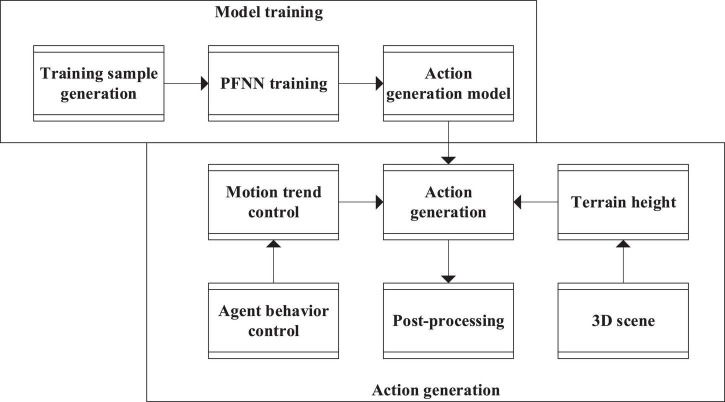
Agent action generation scheme based on the Phase Functioned Neural Network (PFNN).

To generate real-time environment-adaptable geometric actions in character animation, researchers have proposed the PFNN to map the control information to the actions of characters as an input. The PFNN model generates a variable with a phase function to represent the motion period as the weight of the regression network of each frame. Once generated, these weights will be used in the neural network (NN) to generate the character pose of the current frame matching the control parameters in real-time. Meanwhile, PFNN also solves the drift problem by postprocessing the feet. To sum up, based on the requirements of real-time, action diversity, and terrain environmental adaptation of role action, this article proposes an agent action generation scheme based on PFNN for biped agent roles. The brief flow of the scheme is shown in Figure 9.

In the PFNN, the network weight of each frame can be calculated through the phase function α. The phase *p* is compared with the parameter β as an input reference, as calculated in Eq. (1):


(1) α=Θ(p;β)

In Eq. (1), Θ can either represent another kind of NN or the Gaussian process. In this study, the cubic Catmull ROM spline curve is selected so that cycle establishment is greatly reduced because the starting control point is the same as the end control point, and the number of parameters is proportional to the number of control points; the spline curve can change smoothly with the input parameter *p*.

Given four control points composed of network weights β = (α_1_, α_2_, α_3_, α_4_), the cubic spline function definition of network weight can be generated from any phase *p*:


(2) $$\begin{array}{c} \Theta \left(p;\beta \right)=\alpha_{\mathrm{k1}}+w\left(\frac{1}{2}\alpha_{\mathrm{k2}}-\frac{1}{2}\alpha_{\mathrm{k0}}\right)\\ \;+w^{2}\left(\alpha_{k0}-\frac{5}{2}\alpha_{k1}+2\alpha_{k2}-\frac{1}{2}\alpha_{k3}\right)\\ \;+w^{3}\left(\frac{3}{2}\alpha_{k1}-\frac{3}{2}\alpha_{k2}+\frac{1}{2}\alpha_{k3}-\frac{1}{2}\alpha_{k0}\right) \end{array}$$

(3) $$w=\frac{4p}{2\pi }$$

(4) $$k_{n}=\left[\frac{4p}{2\pi }\right]+n-1$$

During network model operation, the PFNN must provide the corresponding phase *p* and the input *x* of the NN in each frame. Because the phase can be stored and calculated on the time axis, the phase *P* is adjusted in the interval of 0 ≤ *p* ≤ 2π.

The agent action generation scheme based on PFNN includes the following two parts:

(1) Model training. Many motion capture data are used for model training to obtain the motion generation model.
(2) Action generation. The terrain height is obtained in the 3D scene through the trained action generation model, the motion trend control is obtained from the behavior control module in the agent behavior modeling, and the action generation model is input to obtain the generated action.

## Usability of the Sand Table Game and Its Therapeutic Effect on Mentally Ill Patients

### Evaluation of Usability

This section mainly tests the performance effect of the designed Sand Table game, applies Sand Table game to some respondents, tests their psychological state before and after the game, and analyzes the parameters obtained from the training of the video action generation model. The result corroborates that the application of VIT and IoT has a far-reaching impact on the evaluation standards of FTP education, and the designed Sand Table game has a significant effect on the mitigation and treatment of adverse psychological states. The performance indexes of the designed Sand Table game also meet the needs and can be applied in subsequent psychotherapy.

**FIGURE 10:**
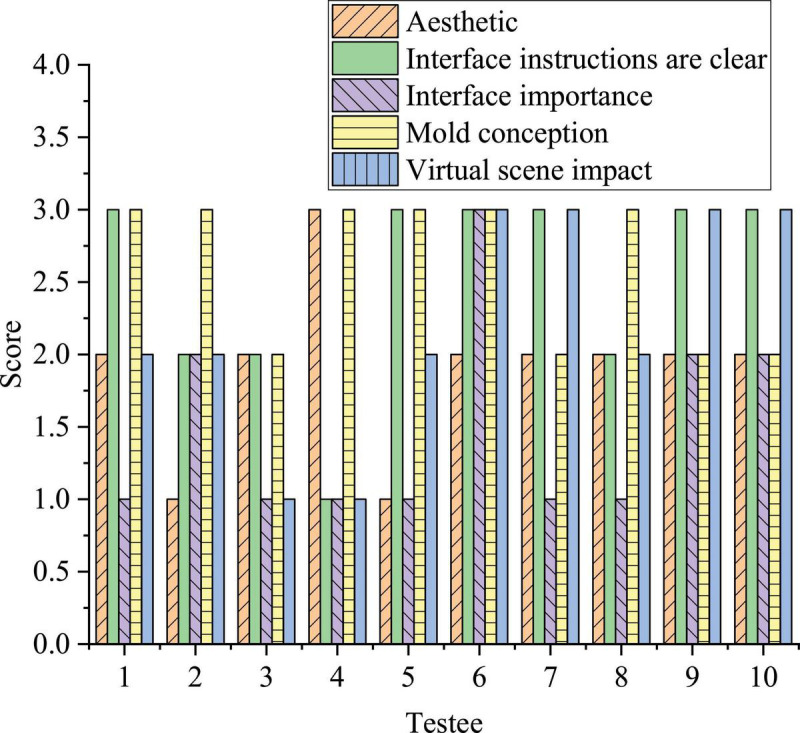
Test results on the usability of VR-based Sand Table game.

Figure 10 indicates the test results of the usability of the VR-based Sand Table game.

Figure 10 indicates that the first testee has rated the clarity of the interface description and the concept of the mold the highest, who thinks that the importance of the interface is low; the second testee has given the highest evaluation on the conceivability of the mold and is not satisfied with the aesthetics of the interface; the third testee has expressed his general disapproval of the game factors; the fourth testee is most satisfied with the aesthetics of the interface and the conceivability of the mold and has not recognized the other three factors; the fifth testee recognizes the clarity of the interface description and the conceivability of the mold but dissatisfies with the importance and beauty of the interface; only the sixth testee has not recognized the aesthetics of the game and has expressed a high evaluation of other factors; the seventh testee has responded well to the clarity of the interface description and the impact of the virtual scene and has not recognized the importance of the interface; the eighth testee is only satisfied with the conceivability of the mold; the ninth and the tenth testees have rated all factors of the game well, especially, the clarity of interface description and the influence of virtual scene. Ten psychology researchers are selected to evaluate the game: among them, 7 subjects felt that the game is generally beautiful, 2 felt that it is very beautiful, and 1 felt that it is not beautiful. Therefore, the beauty setting of the game meets the standard; in terms of the guiding significance of the game interface, 6 subjects feel unclear, 3 feel generally clear, and 1 feel very clear. Therefore, the guiding significance of the game interface needs to be improved, in terms of the importance of the system interface, 6 subjects think it is very important, 3 felt it is generally clear, and 1 felt it is not important. Therefore, the system interface of the game is very important; in terms of the conceivability of the mold, 6 subjects feel that they are conceivable, and 4 feel that they are appropriately conceivable. Therefore, the conceivability of the Sand Table game mold is strong; as for whether the virtual scene has an impact on the audience, two subjects think it has, four think it possibly influences, while four think it definitely does not influence the audience. Therefore, the virtual scene has a strong impact. In short, the respondents have recognized whether the Sand Table mold is beautiful, the importance of the system interface, and the conceivability of the designed Sand Table model.

Therefore, the application of VIT and IoT has a far-reaching impact on the evaluation standards of FTP education.

### Impact on Improving Audience Psychology

**FIGURE 11:**
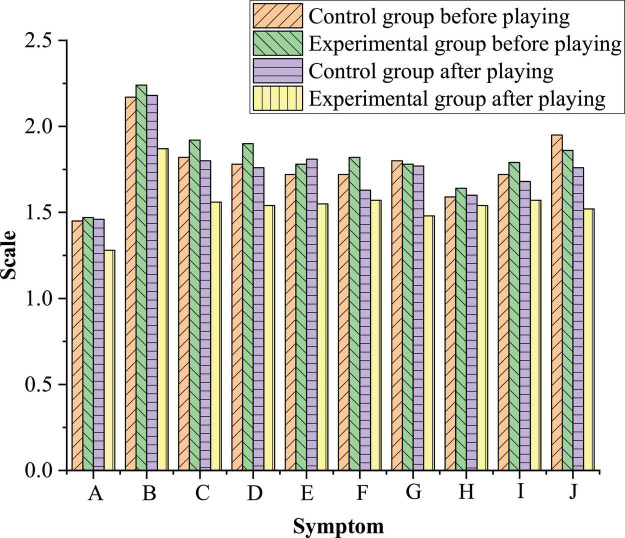
Comparison of bad emotions of students before and after the Sand Table game intervention. (A), somatization; (B), compulsion; (C), interpersonal sensitivity; (D), depression; (E), anxiety; (F), hostility; (G), fear; (H), paranoia; (I), psychosis; (J), other bad emotions or manifestations.

Table 4 compares the TAS scores of respondents before and after they play the designed Sand Table game, and Figure 11 compares the bad emotions of students before and after the game intervention. The experimental group has played the Sand Table game, whereas the control group has not. The experimental group and the control group are tested psychologically separately before and after the designed Sand Table game intervention on the experimental group.

**TABLE 4:** Comparison of the Test Anxiety Scale (TAS) scores before and after the Sand Table game intervention.

Test population		Test number	Total test score
Group	Before the game intervention	62	20.37
	After the game intervention	62	14.15
Control group	Before the game intervention	62	20.37
	After the game intervention	62	19.71

According to Table 4, there is a gap between the scores of the control group and the experimental group before and after the game intervention. After the game intervention, the total TAS score of the experience group has decreased from 20.37 to 14.15, while the TAS score of the control group has decreased from 20.37 to 19.71, indicating that the Sand Table game can mitigate bad psychology.

Figure 11 illustrates that the control group does not receive any form of psychotherapy, resulting in no significant change in the total TAS scores and the individual symptom score, and the total TAS score has only reduced 0.66 points, which can be the deviation during a normal test. Specifically, the TAS scores of the experimental group fluctuate radically: the score of somatization has decreased 0.5 points, by 24.7%; similarly, the score of compulsion has decreased 0.37 points, by 25.1%; interpersonal sensitivity 0.36, by 18.8%; depression 0.36, by 18.9%; anxiety 0.23, by 12.9%; hostility 0.25, by 13.7%; fear 0.3, by 16.9%; and psychosis 0.22, by 12.3%. Particularly, paranoia has decreased only 0.1 points, with a slight decline. Other bad emotions or performances have decreased 0.45 points, by 24.2%. While, the TAS scores of the control group have changed slightly: somatization score has increased by 0.01 points and 0.6%; compulsion score has increased by 0.01 points and 0.4%; interpersonal sensitivity score has decreased by 0.02 points and 1%; depression score has decreased by 0.02 points and 1.1%; anxiety score has increased by 0.09 points and 5%; hostility score has decreased by 0.09 points and 5%; fear score has decreased by 0.03 points and 1.6%; paranoia score has increased by 0.01 points and 0.6%; mental diseases score decreased by 0.04 points and 2.3%; and other bad emotions or performance score has decreased by 0.19 points and 9.7%. This shows that the Sand Table game has a significant effect on the alleviation and treatment of bad mental states.

### Training Parameter Results of Video Action Generation Model Based on the Phase Functioned Neural Network

The parameter results obtained from the training of the video action generation model are shown in Table 5.

**TABLE 5:** Optimal setting parameters.

Parameter type	Learning rate	Data batch size	Iterations	Number of exit values	Weight decay rate	Number of expert networks
Best training value	0.0001	30	120	0.8	0.003	5

After multiple model training, the optimal setting parameters are obtained as shown in Table 5, and the future trajectory is obtained by prediction. When preparing to run and input the initial × position, it is necessary to get the action gait label through the motion control parameters of the agent, enable the required actions, such as sitting, standing, idle, lying down, jumping, and moving, and get the speed and direction of the character. The target velocity and direction are interpolated smoothly and finally used to predict the future trajectory. The exponentially weighted deviation is used to infer the trajectory curve, which defines the maximum length of the future trajectory, thereby providing the required character speed in m/s and producing a smooth trajectory. This constitutes all the required input variables, and the output position can be calculated. In terms of terrain adaptation, different from the scheme of terrain matching and training of data in the action generation model of biped character, the output motion is simply processed by whole-body reverse dynamics, including the processing of foot nodes, so that their motion can adapt to the height change of terrain, and the motion of spine nodes is updated relative to the height of the terrain. This can avoid the stretching effect of limbs when going uphill and downhill, thus getting more natural and realistic movements.

## Discussion

The results obtained in this article are summarized as follows. The designed VR-based Sand Table game carried on IoT technology aims to improve the therapeutic effect of mentally ill patients. After a series of experiments, first, the beauty of the designed Sand Table game has been recognized to a certain extent. Moreover, Kao et al. (2021) believed that VR had subverted the game market and was rapidly popularizing. The difference between VR and traditional media (such as controllers visible in the virtual world) made new teaching methods possible. They proposed a VR game using text + chart and text + space, which significantly improved the learnability and performance of controls. It showed that the game type was a key factor in tutorial mode design (Kao et al., 2021). Similarly, this study also holds that VR technology can be applied in daily life. Apparently, the designed Sand Table game model is easy to solve, and the further appearance can be optimized in the follow-up study. In the following experiments, the therapeutic effect of the designed Sand Table game model on mentally ill patients is greatly improved to encourage patients to express their intrinsic feelings through game playing, which is convenient for the treatment work of doctors. Boldi and Rapp (2021) proposed that a commercially successful video game could play a positive role in the treatment of mentally ill patients in the next few years. According to the conclusion of this article, it is agreed that games can have a positive impact on various mental health conditions, and the game-based intervention measures have been gradually and successfully implemented in the field of mental health (Wu W. et al., 2020; Boldi and Rapp, 2021).

## Conclusion

First, the types and trendy development of mental diseases are analyzed from a global and domestic perspective, and the combination of VIT and IoT technology is explored under the intelligent media era. Then, the Sand Table game is designed by integrating VR and IoT technology, and the Sarason TAS is introduced. Then, the VR-based Sand Table game is improved, and through QS analysis, the therapeutic effect of the designed Sand Table game is tested. The experimental results suggest that the designed Sand Table game has complete usability, perfect PP process, and plays a significant role in alleviating and treating the mental problems of the audience. Many studies believe that the Sand Table game is excellent psychological therapy, as well as a psychological projection test to evaluate mental conditions of patients without demanding specific conclusions; moreover, the gaming process is regarded as either an expression reflecting the internal world of patients or their communication with their therapists who appears to be idlers while actually trying to communicate with the patients in-depth and empathize with intrinsic emotions of patients and understand the complex psychological expression behind these emotions. Sand Table game is suitable for both students and patients with mental problems with no regard to age, which can be used to treat anxiety, depression, and other mental problems. The research purpose has been achieved by analyzing the impact of the VIT and IoT technology on the FTP education of college students and the audience psychology. In brief, the designed VR-based Sand Table game can help players manipulate props more flexibly within a virtual environment, while the IoT + VR technology can improve the traditional Sand Table game by allowing therapists to treat patients online, so patients might choose therapeutic time freely and without worrying too much about geographic distances; meanwhile, therapists can share Sand Table game with many treatment institutions and educational institutions over the network to provide more perfect treatment processes for mentally ill patients. Specifically, the results read as follows: the first experiment shows that the designed Sand Table game model has high aesthetics and has been highly praised by the respondents, but it also needs some correction and beautification; the second experiment verifies that the designed VR-based Sand Table game model can show the internal world of patients through model shaping, provide better therapeutic effects, and have a certain psychological counseling effect on mentally sub healthy people; the last experiment implies that the motion authentication performance of the model is high, and the most suitable areas of patients can be judged according to the motions of patients. Although research objectives have been achieved and valuable research conclusions have been achieved, some deficiencies have not been avoided. For example, (1) this experiment does not select sufficient patients to cover the wide variety of mental diseases; (2) there is a need to design a Sand table game that can show distinct effects on different states of mental diseases; (3) the game functions of VR-based Sand Table game need to be optimized in terms of beauty and fineness. These two aspects will be the focus of the follow-up study.

## Data Availability Statement

The raw data supporting the conclusions of this article will be made available by the authors, without undue reservation.

## Ethics Statement

The studies involving human participants were reviewed and approved by the Ethics Committee of Sichuan University of Media and Communications. The patients/participants provided their written informed consent to participate in this study. Written informed consent was obtained from the individual(s) for the publication of any potentially identifiable images or data included in this article.

## Author Contributions

All authors listed have made a substantial, direct, and intellectual contribution to the work, and approved it for publication.

## Conflict of Interest

The authors declare that the research was conducted in the absence of any commercial or financial relationships that could be construed as a potential conflict of interest.

## Publisher’s Note

All claims expressed in this article are solely those of the authors and do not necessarily represent those of their affiliated organizations, or those of the publisher, the editors and the reviewers. Any product that may be evaluated in this article, or claim that may be made by its manufacturer, is not guaranteed or endorsed by the publisher.

## Appendix

**TABLE A1:** Sand table game mental health QS.

Type of information	Basic information
What is the research duration of psychotherapeutic Sand Table Game	1. 1–3 years 2. 4–7 years 3. 8–10 years
Gender	1. Male 2. Female
Do you know about Sand Table Games or other kinds of Sand Table Games?	1. Yes 2. No
Do you think the designed game is in line with the theoretical basis of the Sand Table Game?	1. Exactly consistent 2. Relatively consistent 3. Not consistent
Do you think the principle analysis of the Sand Table Game is correct?	1. Absolutely right 2. Quite right 3. Not right
Do you think the game rule design of the system is reasonable?	1. Very reasonable 2. Reasonable 3. Unreasonable
Do you think Sand Table Game molds be conceptive?	1. Surely conceptive 2. Appropriately conceptive 3. Completely conceptive
What do you think is the most important application mode of the system?	1. Single 2. Group
What do you think is the most valuable usage mode of the system?	1. Same place at the same time 2. Same place and different time 3. Different place at the same time 4. Different place at different time
What do you think of the system interface aesthetics	1. Beautiful 2. Generally beautiful 3. Unbeautiful
Do you think the guiding significance of the system interface is clear?	1. Very clear 2. Generally clear 3. Uncertain
What do you think of the importance of the system interface?	1. Very important 2. Important 3. Unimportant
Do you think the 3D SPT model meets the standard?	1. Very consistent 2. General consistent 3. Not consistent at all
Do you think the virtual scene will affect the visitors?	2. Will 2. Probably will 3. Will not
Do you think the system can be used for psychological SPT?	1. Can 2. Probably can 3. No
Do you think the proposed system is worthy of further research and promotion?	1. Extremely worthy 2. Generally worthy 3. Unworthy
Do you think the system is difficult to get started with?	1. Very difficult 2. Difficult 3. Easy
What do you think of the difficulty of completing the assigned task?	1. It’s very difficult for the system to complete 2. Generally simple, and the system can complete the tasks 3. Difficult, and the system can barely be realized 4. Very simple and the system implementation is more than enough
Other supplements	
